# Voice-related quality of life and emotional symptoms before and after thyroidectomy

**DOI:** 10.1590/2317-1782/20212021118

**Published:** 2022-02-02

**Authors:** Gustavo Batista de Oliveira, Thais Jejesky de Oliveira, Marco Homero de Sá Santos, Ricardo Mai Rocha, Michelle Ferreira Guimarães, Elma Heitmann Mares Azevedo

**Affiliations:** 1 Departamento de Fonoaudiologia, Universidade Federal do Espírito Santo – UFES - Vitória (ES), Brasil.; 2 Hospital Universitário Cassiano Antônio Moraes – EBSERH - Vitória (ES), Brasil.; 3 Departamento de Cirurgia de Cabeça e Pescoço, Universidade Federal do Espírito Santo – UFES - Vitória (ES), Brasil.

**Keywords:** Voice, Thyroidectomy, Quality of Life, Anxiety, Depression

## Abstract

**Purpose:**

To correlate voice-related quality of life, anxiety, and depression symptoms pre- and post-thyroidectomy.

**Methods:**

Observational, longitudinal, prospective, and quantitative study. Twenty patients participated in the study, with a mean age of 54 years, who underwent thyroidectomy, laryngeal visual examination, and the Voice-Related Quality of Life and Hospital Anxiety and Depression Scale questionnaires at different times: preoperative, 1 week and 3 months post-thyroidectomy, with a higher prevalence of females (85%; n=17) and partial thyroidectomy (70%; n=14).

**Results:**

There was no statistical difference in voice quality of life between the moments, but lower preoperative scores were observed, especially in the physical domain. We observed a slight trace of anxiety in the preoperative period, with a reduction after 1 week and an increase after 3 months. There was a moderate negative correlation between the physical domain of QVV and the anxiety subscale and the total HADS score after 1 week and between the total domain of QVV with the total HADS score after 1 week, weak negative correlation between the total domain of QVV and the HADS anxiety subscale after 1 week and the total HADS score after 3 months.

**Conclusion:**

The patients evaluated in this study self-perceived their voice-related quality of life as positive. Mild anxiety traits were identified, with a reduction after one week postoperatively and an increase after three months. The self-perception of better voice-related quality of life in the postoperative period is weakly related to the reduction of anxiety levels.

## INTRODUCTION

Thyroid diseases affect hormone production and secretion^(1)^. They can alter vocal outcomes and deglutition^(2)^ to varying degrees and at different moments of surgical treatment, according to individual clinical-pathological factors, even in the absence of neural injuries. They also increase public health costs^(1-7)^.

The impacts of thyroidectomies have improved over time^(3)^, however, in addition to functional aspects there can be negative impacts on voice-related quality of life, with reductions during the pre-operative period^(2,6)^, and emotional alterations^(3,8-10)^ associated with the diagnosis of thyroid nodules.

Although the influence of thyroid function on mood alterations is little understood, there is evidence that thyroid alterations lead to emotional ones^(3,8)^. Some studies report a high prevalence of anxiety and depression in patients with thyroid disturbances^(8,9,11)^, with the waiting period for surgery and the weight of the diagnosis being determinant on patient anxiety and depression ^(12)^.

The literature related to emotional aspects and to voice-related quality of life in individuals who undergo thyroidectomies is scarce and understanding such parameters is important when making clinical decisions^(2)^, during follow up with patients and in their ongoing therapeutic planning. According to Koga et al.^(2)^ women with thyroid disease, show negative impacts on voice-related quality of life during the pre-operative period, mainly in the physical domain. The authors highlight that, to the extent that physical events related to the voice occur, there is also an impact on the socioemotional dimension.

Thus, the present study seeks to correlate the voice-related quality of life and symptoms of anxiety and depression in pre-operative and immediately post-operative (maximum of 1 week) patients, and 3 months post-thyroidectomy. The follow-up period was chosen based on previous studies that reported the presence of vocal symptoms over the short and long term, expecting that, at around 3 months, there is a reduction with the possibility of long-term persistence^(13,14)^.

## METHODS

The present study is observational, longitudinal, prospective, and quantitative. It was approved by the Research Ethics Committee of the Institute of origin, (process number 2.930.664). All individuals who participated in the study signed the Free and Informed Consent Form.

Twenty patients with thyroid disease were included, with a higher prevalence of females (85%; n=17), partial thyroidectomy (70%; n=14), and an average age of 54 years (± 16.9). Patients were attended at the Head and Neck Surgery Ward of the University Hospital. The exclusion criteria were laryngeal alterations and endolaryngeal signs of laryngopharyngeal reflux, observed via videolaryngoscopy, with the presence of hyperemia and edema of the posterior third of the glottic and interarytenoid regions. The data for thyroidal hormone alterations were obtained through TSH dosage (thyroid stimulating hormone) and free T4 in the blood. It was solicited in a regular manner in the clinical follow-ups with patients and consistently tabulated from the medical records.

Patients were evaluated using the Voice-Related Quality of Life Questionnaire (QVV) and the Hospital Anxiety and Depression Scale – HADS. The QVV evaluates voice-related quality of life using self-perception and contains 10 statements divided into three domains: socio-emotional (questions 4, 5, 8 and 10), physical (questions 1, 2, 3, 6, 7 and 9) and global (all the questions). The answers varied on a scale from 1 to 5, wherein 1 equaled “never occurs and it is not a problem” and 5 equaled “it always occurs and it really is a bad problem”. The total score varied from 0 to 100 (from the worst to the best quality of life) and there is a score for each domain, according to the formula for calculation of the protocol^(15)^. The cut-off score is 91.25 points and the averages for the global domain observed for dysphonic individuals and individuals with healthy voices were 65.9 and 98 points, respectively^(16)^.

The HADS scale is used to evaluate mood disorders in patients with physical diseases, containing 14 multiple choice questions, divided into two subscales, one for anxiety and the other for depression, with each one containing seven items. The overall score for each subscale varies between zero and 21. The scores for anxiety and depression are categorized as normal (0-7), slight (8-10), mild (11-14) and severe (15-21). The cutoff point of 8 or more points identifies possible cases of anxiety/depression, and 11 or more, probable cases of anxiety/depression^(17,18)^.

For data analysis, descriptive statistics of the variables studied were realized. The Friedman Test was used for comparison of the pre- and post-surgery moments and the Conover Post-hoc Test to precisely identify during which moments of the study the differences occurred. To measure the correlation between the scoring of the instruments, the Spearman Correlation Test was used. A significance level of 5% was adopted (p-value ≤ 0.05). Correlations below 0.50 were considered weak, between 0.50 and 0.7 moderate, between 0.70 and 0.90 strong and above 0.90 very strong^(19)^. All statistical analyses were realized on the R program, version 3.6.1.

## RESULTS

A statistically significant difference in the voice-related quality of life between the different moments was not observed, although self-perception of QV in the voice was worse during the pre-operative period, with an emphasis on the physical domain in the different moments (Table 1).

**Table 1:** Comparison of scores of the QVV domains during the different moments in patients submitted to thyroidectomy

**QVV**	**Moment**			**p-value**
	**Pre**	**Post 1W**	**Post 3M**	
Socioemotional	75.9	86.2	89.1	0.705
Physical	75.0	77.1	82.9	0.768
Total	75.4	80.8	85.4	0.907

**Caption**: QVV= Voice-Related Quality of Life; Pre= pre-operative; Post- 1W= post one-week; Post- 3M= post three-months

Using the HADS, a statistically significant difference between the scores in the three moments was observed for all domains, with the greatest difference observed between the pre-operative moment and 1 week after surgery. There were very high values for slight traces of anxiety during the pre-operative period, which were more frequent in comparison with depression for all moments, with a reduction between the pre- and post- 1 week moments and an increase between the post- 1 week and the post three-months after surgery moments, although characterized as “normal trace” (Table 2).

**Table 2:** Comparison of the scores of the HADS domains during the different moments in patients submitted to thyroidectomy

**HADS**	**Moment**			**p-value**
	**Pre**	**Post 1W**	**Post 3M**	
Anxiety	8.1	5.0	6.7	0.012**
Depression	5.4	3.7	4.1	0.010**
Total	13.5	8.7	10.8	0.014**

**Caption**: Friedman Test (**p ≤ 0.05;); HADS= Hospital Anxiety and Depression Scale; Pre= pre-operative; Post 1W= post one-week; Post 3M= post three-months

Applying the Post-hoc Conover Test and comparing the moments in pairs, a statistically significant difference was observed between all pairs of moments for the Anxiety domain and the total HADS score. In the Depression domain, there was a statistically significant difference between the pre- and post- 1 week and 3 month moments (1W/3M) (Table 3).

**Table 3:** Multiple comparisons of the scores of the HADS domains during the different moments in patients submitted to thyroidectomy

**HADS**	**Moment**		
	**Pre/Post 1W**	**Pre/Post 3M**	**Pre 1W/ Post 3M**
Anxiety	< 0.001**	< 0.001**	< 0.001**
Depression	< 0.001**	< 0.001**	0.5
Total	< 0.001**	< 0.001**	< 0.001**

**Caption:** Conover post-hoc test (**p ≤ 0.05); Pre= pre-operative; Post 1W= Post one-week; Post- 3M= post three-months

There were moderate negative correlations between the physical domain of the QVV with the anxiety subscale and the total HADS score post- 1 week, and between the total QVV domain and the total HADS score post- 1 week. There was a weak negative correlation between the total QVV domain and the anxiety subscale of the HADS post- 1 week and the total HADS score post- 3months (Table 4).

**Table 4:** Correlation between the scores of the QVV domains and the HADS items during the different moments in patients submitted to thyroidectomy

**QVV**	**HADS-A**			**HADS-D**			**HADS-T**		
	**Pre-**	**Post- 1S**	**Post- 3M**	**Pre-**	**Post- 1S**	**Post- 3M**	**Pre-**	**Post- 1S**	**Post- 3M**
Socioemotional	0.086	-0.228	-0.191	-0.106	-0.128	-0.281	0.013	-0.263	-0.287
Physical	0.059	-0.545**	-0.410	-0.123	-0.315	-0.370	-0.015	-0.536**	-0.435
Total	0.086	-0.478**	-0.395	-0.106	-0.318	-0.416	0.013	-0.501**	-0.461**

**Caption**: Spearman correlation test (**p ≤ 0.05); QVV= Voice-related Quality of Life; HADS-A= anxiety domain of the Hospital Anxiety and Depression Scale; HADS-D= depression domain of the Hospital Anxiety and Depression Scale; HADS-T= total domain of the Hospital Anxiety and Depression Scale; pre-= pre-operative; 1W= one-week; 3M= three-months

## DISCUSSION

Seeking to understand the functional results for quality of life and emotional symptoms before and after the realization of thyroidectomies has become a constant and is increasingly present in the literature even in contexts involving the preservation of the laryngeal nerves^(1,2,13,14,20)^. Knowledge of these parameters in this population is of extreme importance for the speech therapy approach both in the pre- and post-operative moments, which are increasingly more individualized and centered on the needs of the patient.

For the QVV questionnaire results, there was no statistically significant difference between the moments, however the lowest scores were self-declared during the pre-operative moment with the physical domain for all moments standing out, and a progressive improvement for all scores after three months. The presence of vocal symptomatology prior to the thyroidectomy^(2,21)^ can affect voice-related quality of life, especially, in the physical domain, and it is expected that with the reduction of vocal symptoms following surgery, there is an improvement in the domains related to quality of life^(6,22)^.

Quality of life is also related to the emotional and social impacts of the surgical thyroid treatments even if the patient presents good general health following treatment^(12)^. According to Koga et al.^(2)^ in the pre-operative moment the physical domain is the most affected, which could be due to the physiological alterations affecting the phonatory system at the vocal or respiratory levels, which can be present prior to the thyroidectomy. Alterations in thyroid gland size as well as malignity can cause compressive symptoms in the laryngotracheal region and explain vocal alterations encountered prior to surgery^(1,23)^.

Regarding the presence of anxiety and depression symptoms, our results showed the presence of slight traces of anxiety, greater than those for depression, with higher values during the pre-operative moment, a reduction between the pre- and post- 1 week moments and an increase between the post one-week and post three-months after surgery moments.

Despite the depression symptoms being within a normal range, a reduction was observed, and subsequently, an increase in the values indicating the need for attention to this symptomatology.

When comparing the moments, a statistically significant difference was observed between all the moments for anxiety and for the total domain of the HADS. However, a statistically significant difference was only observed between the pre- and post- 1W/3M moments in the depression domain, showing the importance of investigation prior to treatment with greater relevance for anxiety, which is sometimes overlooked. This highlights the importance of evaluating this parameter during all treatment moments.

Patients with thyroid diseases awaiting surgery, present high anxiety levels during the pre-operative moment, independent of the severity of the disease or the complexity of the surgery. This can occur due to a lack of information prior to the procedure, as well as due to patient expectations^(9)^. This can diminish after the procedure as seen in this study between the pre and 1W post-operative moments. It is worth noting that in 2019, Geser and Arslan^(24)^ observed that pre-operative guidance with information pamphlets did not alter post-operative anxiety.

The increase in the score for the anxiety trait at 3 months post-surgery could be related to concerns about the risk of future recurrence of thyroid alterations and uncertainty about the treatment^(25)^. However, it is worth noting that, despite the score increasing after 3 months, in our results this score was within normality^(17,18)^. Kim et al.^(26)^ found that patients who undergo thyroidectomies reduce fatigue and anxiety and improve quality of life by undertaking physical activity. In our study, we do not have these data to compare.

According to Choi et al.^(27)^ the incidence of depression increases during the immediate post-operative period following partial or total thyroidectomy, and remains elevated for one to two years, therefore requiring that the patient undergo long term psychological monitoring. Patients with thyroid cancer submitted to thyroidectomy present depressive disorders more frequently. Recuperation from depression is faster in middle-aged or older patients, and those with higher income or resident in rural areas, in comparison with younger patients, with lower incomes or from urban areas^(28)^.

The correlations indicate an inverse association between the two scores. Therefore, the self-perception of better QVV in the voice in the pos-operative moment is related to the reduction in anxiety levels. The relation between vocal and psychological characteristics of dysphonic patients is little considered in the literature. In 2020, Brazilian authors encountered an association between dysphonia and anxiety, recommending group therapy as a strategy to reduce these parameters^(29)^.

It is common for patients during the pre-operative moment to present emotional symptoms, which can be reduced through phonoaudiological guidance and support, increasing the connection with the professional and the commitment of the patient to the therapeutic plan. This justifies the importance of the pre-operative intervention and the need for early phonoaudiological intervention, independent of post-operative complications.

Some study limitations were observed, including the reduced sample size due to the suspension of the clinic during the COVID-19 pandemic, and difficulties with communication by telephone, since some calls were not concluded or the patient could not be reached. Important information regarding the motivations which led patients to be undergo the thyroidectomy were not collected. Additionally, the sample could have been better described with data related to the thyroid disease, which could affect clinical outcomes and enrich the discussion.

## CONCLUSION

Patients evaluated in this study who underwent a thyroidectomy presented a positive self-perception of voice-related quality of life. Slight signs of anxiety were identified, with a reduction at the one-week post-operative moment and an increase at the 3 months post-operative moment. The improved self-perception of QV in the voice in the post-operative moment is related, in a weak manner, to the reduction in anxiety levels.
