# Supplementary material for: Voice-related quality of life and emotional symptoms before and after thyroidectomy
Source: Codas. 2022 Feb 2;34(4):e20210118. doi: 10.1590/2317-1782/20212021118 (PMC9886117; doi:10.1590/2317-1782/20212021118)
Supplement: Translation

# Translation: Qualidade de vida em voz e sintomas emocionais pré e pós-tireoidectomia

**Authors:** Gustavo Batista de Oliveira, Thais Jejesky de Oliveira, Marco Homero de Sá Santos, Ricardo Mai Rocha, Michelle Ferreira Guimarães, Elma Heitmann Mares Azevedo

**Affiliations:** 1 Departamento de Fonoaudiologia, Universidade Federal do Espírito Santo – UFES - Vitória (ES), Brasil.; 2 Hospital Universitário Cassiano Antônio Moraes – EBSERH - Vitória (ES), Brasil.; 3 Departamento de Cirurgia de Cabeça e Pescoço, Universidade Federal do Espírito Santo – UFES - Vitória (ES), Brasil.

**Keywords:** Voz, Tireoidectomia, Qualidade de Vida, Ansiedade, Depressão

## Abstract

**Objetivo:**

Correlacionar a qualidade de vida em voz e sintomas de ansiedade e depressão pré e pós-tireoidectomia.

**Método:**

Estudo observacional, longitudinal, prospectivo e quantitativo. Participaram 20 pacientes, com média de idade de 54 anos, submetidos à tireoidectomia, ao exame visual laríngeo e aos questionários Qualidade de Vida em Voz e Escala Hospitalar de Ansiedade e Depressão nos momentos: pré-operatório, 1 semana e 3 meses pós-tireoidectomia, com maior prevalência do sexo feminino (85%; n=17) e de tireoidectomia parcial (70%; n=14).

**Resultados:**

Não houve diferença estatística quanto à qualidade de vida em voz entre os momentos, porém observaram-se menores escores no pré-operatório, com destaque para o domínio físico. Houve traço leve de ansiedade no pré-operatório com redução após 1 semana e aumento pós três meses. Houve correlação negativa moderada entre o domínio físico do QVV e a subescala de ansiedade e o escore total da HADS pós 1 semana e entre o domínio total do QVV com o escore total da HADS pós 1 semana, correlação negativa fraca entre o domínio total do QVV e a subescala de ansiedade da HADS pós 1 semana e o escore total da HADS pós 3 meses.

**Conclusão:**

Os pacientes avaliados neste estudo autopercebem sua qualidade de vida relacionada à voz como positiva. Traços leves de ansiedade foram identificados, com redução no pós-operatório uma semana e aumento pós três meses. A autopercepção de melhor qualidade de vida em voz no pós-operatório relaciona-se, de forma fraca, com a redução dos níveis de ansiedade.

## INTRODUÇÃO

Doenças da tireóide afetam a produção e a secreção hormonal^(1)^, podem alterar os resultados vocais e de deglutição^(2)^, em diferentes graus e momentos do tratamento cirúrgico, de acordo com fatores clínico-patológicos individuais mesmo na ausência de lesão neural e aumentam os gastos em saúde pública^(1-7)^.

Os impactos da tireoidectomia vêm melhorando com os anos^(3)^, mas além dos aspectos funcionais pode haver impacto negativo na qualidade de vida em voz, com redução no pré operatório^(2,6)^, e alterações emocionais^(3,8-10)^ associadas ao diagnóstico dos nódulos tireoidianos.

Embora o papel da função tireoidiana nas alterações do humor seja pouco conhecido, existem evidências de que alterações tireoidianas levam a alterações emocionais^(3,8)^. Alguns estudos referem alta prevalência de ansiedade e depressão em pacientes com distúrbios da tireoide^(8,9,11)^, com o tempo de espera para realização da cirurgia e o peso do diagnóstico sendo determinantes na ansiedade/depressão do paciente^(12)^.

A literatura é escassa ao relacionar os aspectos emocionais e a qualidade de vida em voz em indivíduos submetidos à tireoidectomia e compreender tais parâmetros é importante nas tomadas de decisão clínica^(2)^, no seguimento do paciente e sua adesão ao planejamento terapêutico. Segundo Koga et al.^(2)^ mulheres com doença tireoidiana, no pré-operatório, apresentam impacto negativo na qualidade de vida em voz, principalmente no domínio físico. Os autores ressaltam que, à medida que os acometimentos físicos relacionados à voz acontecem, há impacto também no domínio socioemocional.

Desta forma, o presente estudo teve como objetivo correlacionar a qualidade de vida em voz e os sintomas de ansiedade e depressão em pacientes no pré-operatório, pós-operatório imediato (máximo 1 semana) e 3 meses pós-tireoidectomia. O tempo de seguimento escolhido foi baseado em estudos prévios que relatam a presença de sintomas vocais no curto e longo prazo, esperando-se que, por volta de 3 meses, haja redução com possibilidade de persistência ao longo do tempo^(13,14)^.

## MÉTODO

Trata-se de um estudo observacional, longitudinal, prospectivo e quantitativo com aprovação do Comitê de Ética e Pesquisa da Instituição de origem, sob o número 2.930.664. Todos os indivíduos que participaram do estudo assinaram o Termo de Consentimento Livre e Esclarecido.

Foram incluídos 20 pacientes com doença tireoidiana com maior prevalência do sexo feminino (85%; n=17), tireoidectomia parcial (70%; n=14) e média de idade de 54 anos (± 16,9). Os pacientes foram atendidos no Setor de Cirurgia de Cabeça e Pescoço de um Hospital Universitário. Os critérios de exclusão foram: alterações laríngeas e sinais endolaríngeos de refluxo laringofaríngeo, visualizados por meio de videolaringoscopia, como presença de hiperemia e edema de terço posterior de região glótica e interarinenoidea, e dados de alterações hormonais tireoidianas obtidas pela dosagem de TSH (hormônio tireoestimulante) e T4 livre no sangue, solicitadas de maneira regular no seguimento ambulatorial dos pacientes e colhidas dos prontuários em todos os momentos.

Os pacientes foram avaliados por meio do questionário Qualidade de Vida em Voz (QVV) e da Escala Hospitalar de Ansiedade e Depressão (*Hospital Anxiety and Depression Scale - HADS*). O QVV avalia a qualidade de vida em voz pela percepção do indivíduo e possui 10 afirmações divididas em três domínios: sócio-emocional (questões 4, 5, 8 e 10), físico (questões 1, 2, 3, 6, 7 e 9) e global (todas as questões). As respostas variam em uma escala de 1 a 5, na qual 1 equivale a “nunca acontece e não é um problema” e 5 a “acontece sempre e realmente é um problema ruim”. O escore total varia de 0 a 100 (da pior à melhor qualidade de vida) e há um escore para cada domínio, de acordo com a fórmula própria de cálculo do protocolo^(15)^. A nota de corte é de 91,25 pontos e as médias do domínio global encontradas para indivíduos disfônicos e com vozes saudáveis foram 65,9 e 98 pontos, respectivamente^(16)^.

A escala *HADS* é usada para avaliar transtornos do humor em pacientes com doenças físicas, contém 14 questões do tipo múltipla escolha divididas em duas subescalas, uma para ansiedade e outra para depressão, com sete itens em cada. A pontuação global em cada subescala varia entre zero e 21. Os escores de ansiedade e depressão são categorizados em normal (0-7), leve (8–10), moderado (11–14) e grave (15–21). O ponto de corte de 8 ou mais pontos identifica casos possíveis de ansiedade/depressão e 11 ou mais casos prováveis de ansiedade/depressão^(17,18)^.

Para análise dos dados, foi realizada estatística descritiva das variáveis estudadas. O Teste de Friedman foi utilizado para comparação dos momentos pré e pós cirurgia e o Teste Post-hoc de Conover para identificar com precisão em quais momentos do estudo ocorreu essa diferença. Para medir a correlação entre as pontuações dos instrumentos foi utilizado o Teste de Correlação de Spearman. Foi adotado nível de significância de 5% (p-valor ≤ 0,05). Correlações abaixo de 0,50 foram consideradas fracas, entre 0,50 e 0,7 consideradas moderadas, entre 0,70 e 0,90 consideradas fortes e acima de 0,90 consideradas muito fortes^(19)^. Todas as análises estatísticas foram realizadas no programa R versão 3.6.1.

## RESULTADOS

Não foi observada diferença estatisticamente significante na qualidade de vida em voz entre os diferentes momentos, embora a autopercepção de QV em voz tenha sido pior autorreferida no pré-operatório, com destaque para o domínio físico nos diferentes momentos (Tabela1).

**Tabela 1:** Comparação dos escores dos domínios do QVV, nos diferentes momentos, em pacientes submetidos à tireoidectomia

**QVV**	**Momento**			**p-valor**
	**Pré**	**Pós 1S**	**Pós 3M**	
Socioemocional	75,9	86,2	89,1	0,705
Físico	75,0	77,1	82,9	0,768
Total	75,4	80,8	85,4	0,907

**Legenda:** QVV = Qualidade de Vida em Voz; Pré = pré-operatório; Pós 1S = pós uma semana; Pós 3M = pós três meses

Por meio da *HAD*S, foi observada diferença estatisticamente significante entre as pontuações nos três momentos, em todos os domínios, sendo a maior diferença observada entre o pré-operatório e após 1 semana de cirurgia, com valores mais altos no pré-operatório para traços leves de ansiedade, os quais foram mais frequente em relação à depressão em todos os momentos, com redução entre o pré e pós 1 semana e aumento entre o pós 1 semana e o pós três meses de cirurgia, embora categorizada como “traço normal” (Tabela 2).

**Tabela 2:** Comparação dos escores dos domínios da *HADS*, nos diferentes momentos, em pacientes submetidos à tireoidectomia

**HADS**	**Momento**			**p-valor**
	**Pré**	**Pós 1S**	**Pós 3M**	
Ansiedade	8,1	5,0	6,7	0,012**
Depressão	5,4	3,7	4,1	0,010**
Total	13,5	8,7	10,8	0,014**

**Legenda:** Teste de Friedman (**p ≤ 0,05;); *HADS* = Escala Hospitalar de Ansiedade e Depressão; Pré = pré-operatório; Pós 1S = pós uma semana; Pós 3M = pós três meses

Por meio do Teste Post-hoc de Conover e comparando os momentos dois a dois, foi observada diferença estatisticamente significante entre todos os pares de momentos no domínio Ansiedade e no escore total da *HADS*. No domínio Depressão houve diferença estatisticamente significante entre os momentos pré e pós 1 semana e 3 meses (1S/3M) (Tabela 3).

**Tabela 3:** Comparação múltiplas dos escores dos domínios da *HADS*, nos diferentes momentos, em pacientes submetidos à tireoidectomia

**HADS**	**Momento**		
	**Pré/Pós 1S**	**Pré/Pós 3M**	**Pós 1S/Pós 3M**
Ansiedade	< 0,001**	< 0,001**	< 0,001**
Depressão	< 0,001**	< 0,001**	0,5
Total	< 0,001**	< 0,001**	< 0,001**

**Legenda:** Teste Post-hoc de Conover (**p ≤ 0,05); Pré = pré-operatório; Pós 1S = pós uma semana; Pós 3M = pós três meses

Houve correlações negativas moderadas entre os domínios físico do QVV com a subescala de ansiedade e o escore total da *HADS* pós 1 semana, e entre o domínio total do QVV com o escore total da *HADS* pós 1 semana. Houve correlação negativa fraca entre o domínio total do QVV e a subescala de ansiedade da *HADS* pós 1 semana e o escore total da *HADS* pós 3 meses (Tabela 4).

**Tabela 4:** Correlação entre os escores dos domínios do QVV e os itens da *HADS*, nos diferentes momentos, em pacientes submetidos à tireoidectomia

**QVV**	***HADS-A***			***HADS-D***			***HADS-T***		
	**Pré**	**Pós 1S**	**Pós 3M**	**Pré**	**Pós 1S**	**Pós 3M**	**Pré**	**Pós 1S**	**Pós 3M**
Socioemocional	0,086	-0,228	-0,191	-0,106	-0,128	-0,281	0,013	-0,263	-0,287
Físico	0,059	-0,545**	-0,410	-0,123	-0,315	-0,370	-0,015	-0,536**	-0,435
Total	0,086	-0,478**	-0,395	-0,106	-0,318	-0,416	0,013	-0,501**	-0,461**

**Legenda:** Teste de Correlação de Spearman (**p ≤ 0,05); QVV = Qualidade de Vida em Voz; *HADS-A* = domínio de ansiedade da Escala Hospitalar de Ansiedade e Depressão; *HADS-D* = domínio de depressão da Escala Hospitalar de Ansiedade e Depressão; *HADS-T* = domínio total da Escala Hospitalar de Ansiedade e Depressão; pré = pré-operatório; 1S = uma semana; 3M = três meses

## DISCUSSÃO

Compreender os resultados funcionais, de qualidade de vida e de sintomas emocionais antes e após a realização de tireoidectomias tem se tornado constante e vem sendo destacado na literatura mesmo diante da preservação dos nervos laríngeos^(1,2,13,14,20)^. É de extrema importância o conhecimento destes parâmetros, nesta população, para a abordagem fonoaudiológica tanto no pré quanto no pós-operatório ser cada vez mais individualizada e centrada nas necessidades de cada paciente.

Quanto ao resultado do questionário QVV, não houve diferença estatisticamente significante nos diferentes momentos, porém os menores escores foram autorreferidos no pré-operatório com destaque para o domínio físico em todos os momentos, e melhora progressiva em todos os escores após 3 meses. A presença de sintomatologia vocal antes da tireoidectomia^(2,21)^ pode impactar a qualidade de vida em voz, sobretudo, no domínio físico, e espera-se que com a redução dos sintomas vocais após a cirurgia, ocorra melhora nos domínios relacionados à qualidade de vida^(6,22)^.

A qualidade de vida está relacionada também aos impactos emocionais e sociais do tratamento cirúrgico da tireóide mesmo que o paciente apresente uma boa saúde geral após o tratamento^(12)^. Segundo Koga et al.^(2)^ no pré-operatório o domínio físico é o mais afetado e isso pode ocorrer pelas alterações fisiológicas relacionadas ao aparelho fonatório, em níveis vocais ou respiratórios, as quais podem estar presentes antes da tireoidectomia. Alterações no tamanho da glândula tireoide e malignidade podem causar sintomas compressivos na região laringotraqueal e justificar alterações vocais encontradas antes da cirurgia^(1,23)^.

Quanto à presença de sintomas de ansiedade e depressão, nossos resultados mostraram presença de traços leves de ansiedade superior aos traços de depressão, com valores mais altos no pré-operatório, redução entre o momento pré e pós 1 semana e aumento entre o momento pós uma semana e pós três meses de cirurgia.

Apesar dos sintomas de depressão estarem dentro da normalidade, observou-se redução e, posteriormente, aumento dos valores indicando a necessidade de atenção a essa sintomatologia.

Ao comparar os momentos foi observada diferença estatisticamente significante entre todos os momentos para ansiedade e para o domínio total da *HADS*, porém no domínio depressão houve diferença estatisticamente significante apenas entre os momentos pré e pós 1S/3M mostrando a importância da investigação antes do tratamento e algumas vezes subvalorizada, com maior relevância para ansiedade destacando a importância de se avaliar tal parâmetro em todas as fases do tratamento.

Pacientes com doenças da tireóide, aguardando pela cirurgia, apresentam alto nível de ansiedade no pré-operatório, independente da gravidade da doença e complexidade da cirurgia, o qual pode ocorrer por falta de informação antes do procedimento e expectativas futuras^(9)^, podendo reduzir após o procedimento como visto neste estudo entre os momentos pré e pós-operatório de 1 semana. Vale ressaltar que em 2019, Geser e Arslan^(24)^ observaram que a orientação pré-operatória por meio de folhetos não alterou a ansiedade no pós-operatório.

O aumento do escore de traço de ansiedade 3 meses pós-cirurgia pode estar relacionado com preocupações futuras quanto ao risco de recorrência da alteração tireoidiana e incertezas quanto ao tratamento^(25)^. No entanto, vale destacar que, apesar do escore aumentar após 3 meses, em nossos resultados o mesmo estava dentro da normalidade^(17,18)^. Para Kim et al.^(26)^ pacientes submetidos à tireoidectomia reduzem a fadiga e ansiedade e melhoram a qualidade vida quando realizam atividade física. No nosso estudo não temos estes dados para relacioná-los.

Segundo Choi et al.^(27)^ a incidência de depressão aumenta no pós-operatório recente da tireoidectomia parcial ou total e permanece elevada de um a dois anos, necessitando, portanto, que o paciente tenha acompanhamento psicológico no longo prazo. Pacientes com câncer de tireoide submetidos à tireoidectomia apresentam transtorno depressivo com mais frequência e a recuperação da depressão é mais rápida em pacientes de meia-idade ou idosos, de alta renda e residentes na zona rural, em comparação com pacientes mais jovens, de baixa renda e urbanos^(28)^.

As correlações indicam associação inversa entre as duas pontuações, portanto, a autopercepção de melhor QV em voz no pós-operatório relaciona-se com a redução dos níveis de ansiedade. A relação entre as características vocais e psicológicas de pacientes disfônicos é pouco abordada na literatura. Em 2020, autores brasileiros encontraram associação entre disfonia e ansiedade, recomendando terapia de grupo como uma estratégia para a redução destes parâmetros^(29)^.

É frequente pacientes no pré-operatório apresentarem sintomas emocionais, os quais podem ser reduzidos por meio das orientações e suporte fonoaudiológico, aumentando o vínculo com o profissional e a adesão do paciente ao planejamento terapêutico. Tal fato justifica a importância da intervenção no pré-operatório e a necessidade de intervenção fonoaudiológica precoce, independente de complicações no pós-operatório.

Foram observadas algumas limitações no estudo, sendo elas o tamanho reduzido da amostra devido à suspensão do ambulatório pela pandemia por COVID-19, a dificuldade de comunicação por telefone, pois algumas chamadas não eram concluídas ou o paciente não era encontrado. Não foram colhidas informações importantes sobre os motivos pelos quais os pacientes foram submetidos à tireoidectomia. Além disso, a amostra poderia ter sido melhor detalhada com dados referentes à doença tireoidiana, os quais podem refletir em implicações clínicas e enriqueceriam a discussão.

## CONCLUSÃO

Os pacientes submetidos à tireoidectomia e avaliados neste estudo apresentaram autopercepção positiva da qualidade de vida relacionada à voz. Traços leves de ansiedade foram identificados, com redução no pós-operatório uma semana e aumento pós três meses. A melhor autopercepção da QV em voz no pós-operatório relaciona-se, de forma fraca, com a redução dos níveis de ansiedade.
